# The impact of emotion management ability on learning engagement of college students during COVID-19

**DOI:** 10.3389/fpsyg.2022.967666

**Published:** 2022-08-05

**Authors:** Xiaochun Lei

**Affiliations:** School of Liberal Arts, Nanjing University, Nanjing, China

**Keywords:** emotion management ability, psychological safety, self-efficacy, learning engagement, COVID-19

## Abstract

During the COVID-19, the wanton spread of novel coronavirus had a huge negative effect on the emotions of college students, resulting in a serious impact on the daily learning behavior of many college students. In this context, college students’ emotion management ability is particularly important. Therefore, based on the results of a questionnaire survey of 580 college students, the present study conducts an in-depth analysis of the relationship between current college students’ emotion management ability and learning engagement, and explores the mediating role of psychological safety and self-efficacy in the relationship between emotion management ability and learning engagement. The results show that college students’ emotion management ability is significantly positive related to learning engagement, psychological safety and self-efficacy; Psychological safety and self-efficacy can play a partial mediating role between emotion management ability and college students’ learning engagement. The results reveal the importance of good emotion management ability of college students during the COVID-19, and enlighten colleges and universities to actively pacify students’ emotions to promote their normal learning.

## Introduction

At the beginning of 2020, the COVID-19 broke out worldwide, which had a great impact on the daily lives of people all over the world. The COVID-19 has become the largest public health emergency in the world. This sudden disaster has posed a major threat to the lives and health of people around the world, but it is also a severe test for the Chinese nation (Jing and Ge, 2021). Governments of various countries have taken many administrative prevention and control measures to deal with the local COVID-19, such as maintaining social distance, home isolation, travel restrictions, etc., in order to reduce the speed of the spread of novel coronavirus.

University campus management and control measures are an important part of the prevention and control measures taken by the entire social system in response to the epidemic. In order to prevent and control the epidemic, the Chinese Ministry of Education has put forward the requirement of “stopping classes without stopping teaching, and stopping classes without stopping learning,” and adopts a combination of government-led, college-based, and social participation to jointly implement and ensure the online teaching activities of colleges and universities during the epidemic prevention and control period (Zhang, 2021). However, it should be noted that college students are at a special period of physical and mental development, and are at a critical stage of forming their outlook on life, values and worldview. The outbreak of major epidemics (i.e., COVID-19) and other social life stressful events not only have a serious impact on the lives of college students, but also cause great psychological pressure on them and reduce their psychological safety, and thus affecting their learning status. In particular, when the epidemic has not been completely controlled, Chinese college students spend most of their time completing online learning tasks through mobile phones and computers software. At the same time, they are not allowed to enter and exit the school at will. With the passage of time and the extension of online classes time, the engagement of some students in online learning tends to decline significantly, and they are less motivated to answer questions (Yan and Yuan, 2020). Thus, in the context of the rapidly changing social environment and the normalization of epidemic prevention and control, how to maintain and improve college students’ learning engagement has become an important element that needs urgent attention in current research.

However, while the epidemic crisis brings a certain amount of psychological stress to college students, it also inevitably has an negative impact on their emotions. On the one hand, college students are bombarded with all kinds of epidemic-related information when browsing traditional news media, Weibo and other we media, which will affect their psychological activities. On the other hand, college students need to adapt to the new online teaching methods and complete various learning tasks arranged by the school. For college students who have been in stressful emotions for a long time, negative emotional states such as loneliness, anxiety, frustration and fear will appear (Fan et al., 2021). If the negative emotions of college students are not paid attention to and adjusted in time, it may bring more problems and obstacles to the psychological condition of college students. The existing literature has analyzed the factors influencing college students’ learning engagement from different perspectives, and these factors include cognitive and behavioral elements (Fredricks et al., 2004). In terms of cognition, scholars have examined the influence of learning motivation and learning achievement on learning engagement, showing that the stronger the learning motivation, the higher the learning engagement; achievement goal orientation can directly predict college students’ learning engagement (Karimi and Sotoodeh, 2019). At the same time, college students’ time insight can also promote college students’ learning engagement (Fredricks et al., 2004). In terms of behavior, scholars have examined the effectiveness of individual physiological factors, learning persistence, and environmental factors in promoting learning engagement (Fredricks et al., 2004; Lu et al., 2022). For college students, as mentioned earlier, while paying attention to the epidemic and academics, college students should also take the initiative to improve their emotion management ability and enhance their sense of self-efficacy, so as to relieve greater psychological stress, better engage in learning activities and reduce the emergence of other negative emotions. Existing studies have not focused on the effect of emotion management ability on learning engagement and its influencing mechanism. In view of this, the present study intends to investigate the relationship between college students’ emotion management ability and learning engagement, and analyze the mediating role of two psychological states, that is, self-efficacy and psychological security, in the relationship between emotion management ability and learning engagement. Thus, we aim to provide empirical data for the research of college students’ learning engagement, and to put forward targeted suggestions for how colleges and universities can alleviate college students’ negative emotions and enhance their learning engagement.

## Theoretical background

### Emotion management ability

Emotion has an important impact on the mental health of college students. Emotion is an important driver of individual behavior and affects the direction of cognitive activity, the choice of behavior, the formation of personality, and the handling of interpersonal relationships. Therefore, it is very important for every individual to manage their emotions well. An individual’s emotional state signifies the individual’s response to the environment and the biological motivational state in adapting to changes of the environment. The emotion behind the behavior is not only an expression of the results of the behavior, but also represents some kind of adaptive motivational factor (Fredrickson, 2001; Fredrickson and Branigan, 2005). Positive emotions and negative emotions have different psychosocial functions, with positive emotions having a positive effect and negative emotions having a negative effect. Therefore, individuals need to manage their emotions and perform the positive function of positive emotions. Emotion management ability is a kind of psychological characteristic, which is the ability to recognize, monitor, and drive one’s own emotions, as well as the ability to recognize and respond appropriately to surrounding situations (Zhang et al., 2022). College students who are in the transition period from susceptibility to stability in psychological development are characterized by bipolarity in positive and negative emotions, tension and relaxation, excitement and calmness. In the process of promoting the all-round development of college students, the guidance, control and regulation of emotion has become an important part. The psychological and educational communities have clearly understood that the cultivation of emotional management ability of college students is an important issue related to the adaptation to society, survival and development of college students. Emotion management ability refers to the ability to correctly identify one’s own emotions and those of others, and to guide, adjust, and control them purposefully, so as to achieve healthy development (Du et al., 2007; Zhang et al., 2022). Research on emotion management has been one of the research hotspots of psychologists at home and abroad, and the theory of emotion management has been exploring the influencing factors between emotion and behavior as the core of its research.

### Psychological safety

Psychological safety refers to the anticipation of possible physically and psychologically related dangers or risk factors, and the individual’s sense of power/powerlessness to cope with these dangers and risk, mainly manifested in the form of a sense of certainty and controllability. Psychological safety is also a “feeling of confidence, safety, and freedom freed from fear and anxiety, especially with regard to the feeling of the satisfaction of one’s present (and future) needs,” which is closely related to the objective situation in which an individual or group lives, and is expressed as a sense of psychological safety when the objective situation satisfies the individual’s or group’s internal needs or experiences. In workplace research, psychological safety is the perception that individuals need to take the consequences of interpersonal risks in the work environment (Edmondson, 1999, 2004). The more psychological safety employees feel about the organization, the more they are willing to communicate and share knowledge (Spreitzer et al., 2012; Liao et al., 2022). With the development of China’s economy, the spread of the COVID-19 epidemic, and the intensification of social competition, every Chinese is under tremendous psychological pressure, and one important group is college students. Thus, in recent years, the psychological safety of college students has attracted the attention and focus of all walks of life (Tatiana et al., 2022). According to Maslow, a humanistic psychologist, psychological safety is the most important determinant of mental health. Existing research have pointed out that psychological safety can provide individuals with an organizational atmosphere of mutual trust and respect, which generates incremental psychological resources, so that individuals are more willing to actively engage in work and learning (Yang et al., 2022). For college students, psychological safety is the preconceptions college students have about risk factors related to their bodies or psyches, and the sense of certainty and control they have in dealing with threats. When college students perceive safety, their willingness to engage in self-expression becomes stronger, and they are more willing to share knowledge with others and interact with them frequently, resulting in more work-learning behaviors (Nembhard and Edmondson, 2006; Han et al., 2022).

### Self-efficacy

Self-efficacy refers to the degree to which people feel confident that they can use the skills they have to perform a certain job behavior (Bandura, 1977). General self-efficacy refers to an overall relatively stable sense of competence or self-confidence that individuals exhibit when dealing with challenges in a variety of environments (Luszczynska et al., 2005). Self-efficacy is attributed to self-beliefs and is influenced by the individual’s own experiences of success or failure in behavior, alternative experiences, verbal persuasion, and emotional arousal. It is an interaction among environment, behavior, and person to interpret human behavior. Self-efficacy is one of the determinants of learning behavior, emphasizing an individual’s self-confidence in his or her own abilities and having a broad impact on an individual’s cognition, emotions, and behavior (Bandura, 1977; Linnenbrink and Pintrich, 2003). The sense of self-efficacy plays a key role in the generation system of human abilities. The self-efficacy generated by college students in the learning process can help them to build confidence in their academic tasks through a series of organizational and executive measures (Zimmerman, 1995). In the college student population, individuals with high self-efficacy exhibit stronger pressure resistance (Schonfeld et al., 2019; Ye et al., 2021) and higher levels of subjective well-being and mental health. In the study-oriented college student groups, the established research mainly focuses on academic self-efficacy, which is mainly expressed as college students’ subjective judgment of learning behavior and achievement ability, referring to a subjective judgment and ability belief of learners about whether they can have the ability, confidence, and strategies to complete learning tasks, including learning ability self-efficacy and learning behavior self-efficacy. Individuals with high academic self-efficacy have strong learning motivation and good learning ability, are able to make positive efforts and dare to overcome academic difficulties (Chang and Tsai, 2022; Kryshko et al., 2022).

### Learning engagement

Learning engagement is an important indicator of the quality of students’ learning process (Xie et al., 2020) and is highly correlated with students’ learning persistence, academic satisfaction, learning performance, and academic completion (Kuh, 2009). In the past decade, the concept and measurement of learning engagement have been paid more and more attention and focus from researchers and practitioners (Bond, 2020). As a multifaceted structure, learning engagement is defined differently by different researchers according to different research contexts. Schaufeli et al. (2002) first extended job engagement to learning and put forward the concept of “learning engagement,” which refers to the positive and fulfilling mental state associated with learning and includes three dimensions: vitality, dedication, and concentration. Vitality means having outstanding energy and resilience, not easily tired in learning and not afraid of hardship; dedication means having a strong sense of meaning, pride, full of enthusiasm for learning and courageous to challenge; concentration means being fully engaged in learning and being able to feel positive and enjoyable experience. From the perspective of learning activities, Fredricks et al. (2004) considered learning engagement as students’ commitment or dedication to learning activities and concluded that learning engagement includes three dimensions: behavioral engagement, emotional engagement, and cognitive engagement. Behavioral engagement refers to the academic or non-academic activities that an individual participates in while in school; emotional engagement is the positive emotional response of an individual in the face of learning tasks or teachers, classmates, and a sense of belonging to school; and cognitive engagement refers to the cognitive strategies that students use in learning, i.e., their psychological resources. Fredricks et al. (2016) subsequently added social engagement, which is the social interaction between students and their peers or teachers, to the three-dimensional framework. Bond (2020), in terms of measurement methods and influencing factors, pointed out that learning engagement is the degree of energy and effort students put into the learning process, which can be observed and measured through indicators such as learners’ behavior, cognition, and affect, and is influenced by internal and external factors such as teacher-student relationship, student-student relationship, learning activities, and learning environment. It is evident that due to the complexity of learning engagement itself, international researchers have not developed a unified understanding of the concept and framework of learning engagement, which has indirectly led to the diversity of research in this area.

## Research hypotheses

### Emotional management ability and learning engagement

Emotion management is a flexible response or a delayed response based on a specific situation that is socially acceptable or tolerated by the individual when faced with a range of emotional developments (Bolton, 2004; Lively and Weed, 2014; Polizzi and Lynn, 2021). During the COVID-19 epidemic, Chinese universities responded to the national prevention and control requirements by restricting students from entering and leaving the campus at will, and even implemented complete closure measures in some places where the epidemic was serious (Zhang, 2021). This tends to lead to emotional instability among college students, which reduces college students’ engagement in learning. However, learning engagement is a student’s commitment or dedication to learning activities and is considered to include behavioral, emotional, and cognitive engagement. Therefore, students with high emotion management ability are able to keep themselves extremely emotionally well in any situation. Emotions affect college students’ motivation to learn (Arguel et al., 2019). When a college student is in a positive emotional state, he/she will become willing to learn, good at learning, and will have a strong interest in learning (Finch et al., 2015). It can be said that good academic emotion is the key to improve college students’ commitment to learning. In today’s world of lifelong learning, it is very important to cultivate good academic emotions in college students so that they can learn actively. At the same time, good emotion makes the function of all the systems and organs of college students more coordinated and sound, which will make them more passionate and creative about learning, and more powerful to overcome the frustration and difficulties in learning (Healey, 2017). Therefore, we hypothesize the following:

Hypothesis 1. Emotional management ability is positively related to college students’ learning engagement.

### The mediating role of psychological safety

According to the crisis-growth model, individuals in a supportive environment have access to more resources to cope with stress and reap more security at the same time. In context of the the COVID-19 outbreak changing people’s lifestyles, college students face a series of psychological shocks in their studies, and these shocks enhance the negative emotions of the college student population, leading to a decrease in their psychological safety (Yan and Yuan, 2020). According to emotional intelligence theory, the ability to regulate emotions, i.e., emotion management, is reflected in the adaptive regulation and control of emotions of self and others. For college students during the epidemic, individuals who can regulate and control their emotions well can obtain higher levels of stable feelings and their psychological safety will continue to climb (Mayer et al., 2001). That is, emotion management ability can significantly contribute to college students’ psychological safety. First, in the face of the complex social environment and the severe campus epidemic prevention and control policies, college students’ emotion fluctuation will aggravate the inner insecurity. And appropriate regulation and management of college students’ emotion can enable them to maintain a good state of mind and increase communication with friends and classmates, which is conducive to improving a greater sense of psychological safety. Second, due to the epidemic prevention and control policies, the contact between students and students and students and teachers has been reduced, which will undoubtedly make students feel scared and panic. Faced with this situation, college students with higher emotion management ability will seem more relaxed and even regulate their emotion through exercise and other means. Compared with individuals with poorer emotion management ability, they are more stable inside, i.e., higher level of psychological safety. Finally, the emotion management ability of college students is mainly reflected in the management ability of their own psychological capital, the essence of which is expressed in how to transform psychological capital into the driving force to promote their own adaptation to environmental changes. In other words, when college students perform self-emotion management, it is a special form of regulating their inner insecurity. Accordingly, emotion management is conducive to improving psychological safety. Therefore, we hypothesize the following:

Hypothesis 2. Emotional management ability is positively related to psychological safety.

From the above analysis, it is easy to find that college students who have higher emotional management ability during the COVID-19 epidemic spread have relatively higher levels of their own psychological safety. However, it should be noted that the sense of psychological safety is a special kind of psychological capital, which is externally expressed as calmness and composure in the face of complex environment. Gong et al. (2012) stated that psychological security represents an environmental state that provides individuals with sufficient certainty and foresight to become more engaged. Edmondson and Lei (2014) stated that psychological security can help individuals overcome anxiety. When individuals have a high sense of psychological security, individuals will spend most of their time on efficiency improvement and goal achievement rather than interpersonal risk prevention, and they will be more inclined to be proactive in presenting themselves and gaining recognition from others. In sum, college students with a higher level of psychological safety are also more likely to devote themselves to their learning and avoid too much disturbance to themselves. In other words, psychological safety is conducive to the daily learning activities of college students. Therefore, based on Hypothesis 2, we hypothesize the following:

Hypothesis 3. Psychological safety plays a mediating role in the relationship between emotional management ability and college students’ learning engagement.

### The mediating role of self-efficacy

Social cognitive theory states that self-awareness and self-regulation play a key role in the formation of self-efficacy, while emotion management ability emphasizes the ability of an individual to perceive and regulate self-emotion and self-emotional states (Bandura et al., 1997). Emotion management ability includes the basic and critical things that individuals should have in coping with the environment, solving problems and adaptive survival. This kind of emotion management ability is highly correlated with individual achievement. Thus, emotion management ability can influence the level of self-efficacy of college students. When college students are faced with the problems such as the impact of the COVID-19 epidemic, academic pressure, and career choice after graduation, they need to cope with the pressure of the environment, study and work requirements by combining environmental needs, their own abilities and personality characteristics. At this time, emotion management ability affects the process of how college students seek relevant information and achieve self-development under environmental pressure. College students with high level of emotion management ability are more likely to cope with these issues smoothly, and successfully dealing with these things implies an increase in the individual’s perceived level of self-efficacy (Chang and Tsai, 2022). In addition, individuals with better emotion management ability will experience fewer negative emotions and have positive self-evaluations on the completion of expected goals and tasks (Wang et al., 2020). Mayer et al. (2001) took emotion management ability as a dimension of emotional intelligence. According to the emotional intelligence model developed by them, individuals with higher emotion management ability are able to control and express their emotions better, and perceive and understand problems and frustrations encountered well, thus promoting rational problem solving, generating internal satisfaction, and gaining more positive emotional experiences, and finally enhancing their own self-efficacy. Gundlach et al. (2003) proposed that the level of emotion management ability affects the level of individual self-efficacy to some extent from the perspective of emotion management ability and causal reasoning model. Therefore, we hypothesize the following:

Hypothesis 4. Emotional management ability is positively related to self-efficacy.

Self-efficacy helps increase college students’ motivation to learn, experience positive emotions, and thus devote more time and energy to learning. College students with higher self-efficacy show more interest in learning, are more likely to use various tools (e.g., online tools) for learning, and are more willing to spend more time on learning (Bassi et al., 2007; Bates and Khasawneh, 2007). They are confident that they can handle difficulties, tend to choose challenging learning tasks, persevere in the face of difficulties, and strive to create conditions to achieve their goals even when the behavior fails to reach them. On the contrary, college students with lower self-efficacy tend to set lower learning goals, have a more negative attitude toward academic challenges, are reluctant to invest effort when frustrated, and have difficulty in mobilizing active learning strategies (Linnenbrink and Pintrich, 2003; Fan and Williams, 2010), they think more about their own shortcomings and imagine the learning task as more difficult when they face the learning task, thus creating more stress, all of which can prevent college students from engaging in high-quality learning engagement. Therefore, based on Hypothesis 2, we hypothesize the following:

Hypothesis 5. Self-efficacy plays a mediating role in the relationship between emotional management ability and college students’ learning engagement.

**FIGURE 1:**
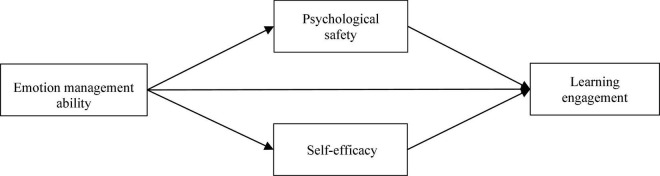
Theoretical model.

Figure 1 shows our theoretical model.

## Materials and methods

### Sample and procedure

We aim to explore how emotion management ability affects college students’ learning engagement, and the mediating roles of psychological safety and self-efficacy. Therefore, the data were collected by means of online research from four universities in northern China in the current study. We collected a total of 650 surveys, and after removing invalid surveys with missing values above 15%, we obtained a total of 580 valid surveys, with an effective rate of 89.23%. The demographic characteristics of the valid sample show that: In terms of gender, 54.6% of participants were male, and 45.4% of participants were female; In terms of grades, 32.4% were freshman, 25.6% were sophomore, 21.7% were junior, 20.3% were senior; In terms of hometown, 65.8% were rural students, 34.2% were urban students; In terms of family background, 21.3% were from divorced family, 78.7% were from non-divorced family; In terms of personality, 29.4% were extroverted, 46.9% were moderate, 23.7% were introverted; In terms of health condition, 89.3% were in good health, 8.7% were in average health, 2% were in poor health.

### Measures

Unless otherwise noted, responses to all items were measured on five-point Likert-type scales, ranging from strongly disagree (1) to strongly agree (5). All variables in this study were measured from well-established scales that are widely used abroad, and all scales have been shown to be valid in Chinese contexts.

#### Emotion management ability

Emotion management ability (EMA) was assessed using a 22-item scale with five sub-scales based on the research of Du et al. (2007). A sample item is “When I encounter something unpleasant, I will find some reasons to comfort myself to reduce the inner disappointment.” In present study, the Cronbach’s α score for this scale was 0.913.

#### Psychological safety

Psychological safety (PS) was assessed using a five-item scale developed by Liang et al. (2012). A sample item is “In my work unit, expressing your true feelings is welcomed.” In present study, the Cronbach’s α score for this scale was 0.804.

#### Self-efficacy

Self-efficacy (SE) was based on a scale developed by Schwarzer et al. (1997) and used 10 items. A sample item is “I believe I can solve problems effectively.” In present study, the Cronbach’s α score for this scale was 0.891.

#### Learning engagement

Learning engagement (LE) was assessed using Fang et al. (2008)’s 17-item scale. A sample item is “I feel energized when I study.” In current study, the Cronbach’s α score for this scale was 0.907.

#### Control variables

In addition, six individual difference variables, including college students’ gender, grades, hometown, family background, personality, and health condition in current study. We controlled them to rule out alternative explanations and to carry out a more reliable test. All the controlled variables were dummy coded. Gender was coded as 1 for participants who were male and 2 for participants who were female. Grades were coded as 1 for participants who were freshman, 2 for participants who were sophomore, 3 for participants who were junior, and 4 for participants who were senior. Hometown was coded as 1 for participants who were rural students, and 2 for for participants who were urban students. Family background was coded as 1 for participants who were from divorced family, and 2 for participants who were from non-divorced family. Personality was coded as 1 for participants who were extroverted, 2 for participants who were moderate, and 3 for participants who were introverted. Health condition was coded as 1 for participants who were in good health, 2 for participants who were in average health, and 3 for participants who were in poor health.

### Data analysis

First, Cronbach’s α, composite reliability, and confirmatory factor an analyses (CFAs) were conducted to assess the reliability and validity of the key variables. Common method variance (CMV) was also assessed. Second, we used hierarchical regression analysis to examine the hypothesized relationships. Finally, we used the bootstrapping method to test mediation because of its high power (Preacher and Hayes, 2004, 2008).

## Results

### Reliability and validity

First, before conducting reliability and validity test, we checked CMV because it is a potential issue in the self-reporting approach research. We used Harmon’ one-factor test by including all of the items of the five variables (i.e., emotion management ability, psychological safety, self-efficacy, and college students’ learning engagement) to examine CMV in SPSS 25.0. When the first emerging factor accounted for over 50% of the extracted variables’ variance, common method bias was suggested and CMV would be a problem. The results demonstrated that the first emerging factor accounted for 27.91% of the explained variance, indicating that CMV was not a significant problem in the present study.

Second, we calculated Cronbach’s α of emotion management ability, psychological safety, self-efficacy, and college students’ learning engagement to examine the reliability. As mentioned above, the values of Cronbach’s α were greater than the threshold value of 0.80, demonstrating acceptable reliability.

Finally, we conducted a series of CFAs using Amos 23.0 on the scales including emotion management ability, psychological safety, self-efficacy, and college students’ learning engagement to examine discriminate validity (see Table 1). Results showed that the fit of the five-factor model in which items were loaded on their respective measures was better than any other model (χ^2^/df = 2.977, RMSEA = 0.062, CFI = 0.911, TLI = 0.909, IFI = 0.911, SRMR = 0.060). These results of CFA provided full support for the discriminate validity of our study instruments.

**TABLE 1:** Results of confirmatory factor analyses.

Models	Variables	χ^2^	df	χ^2^/df	IFI	RMSEA	CFI	TLI	SRMR
Four-factor model	EMA, PS, SE, LE	1,565.902	526	2.977	0.911	0.062	0.911	0.909	0.060
Three-factor model	EMA, PS, SE + LE	2,065.327	523	3.949	0.873	0.092	0.872	0.856	0.085
Two-factor model	EMA, PS + SE + LE	3,150.954	531	5.934	0.742	0.125	0.751	0.725	0.091
One-factor model	EMA + PS + SE + LE	5,495.122	526	10.447	0.534	0.171	0.531	0.486	0.149

### Descriptive statistics and correlations

We calculated the correlations among emotion management ability, psychological safety, self-efficacy, and college students’ learning engagement using SPSS 25.0. As shown in Table 2, emotion management ability was positively related to psychological safety (*r* = 0.334, *p* < 0.01), positively related to self-efficacy (*r* = 0.285, *p* < 0.01), and positively related to college students’ learning engagement (*r* = 0.264, *p* < 0.01). Psychological safety was positively related to college students’ learning engagement (*r* = 0.488, *p* < 0.01). At the same time, self-efficacy was also positively related to college students’ learning engagement (*r* = 0.591, *p* < 0.01). These results provided preliminary supports for the hypotheses proposed above. We further used hierarchical regression analysis and bootstrapping method to test the hypotheses.

**TABLE 2:** Results of correlation analysis.

	1	2	3	4	5	6	7	8	9
1. Gender									
2. Grades	0.001								
3. Hometown	0.002	0.037							
4. Family background	0.001	0.023	0.005						
5. Personality	0.005	0.044	0.068	0.101*					
6. Health condition	0.013	0.057	0.004	0.017	0.020				
7. EMA	0.024	0.013	0.037	0.088	0.035	−0.085			
8. PS	0.035	0.057	0.018	0.064	−0.027	0.042	0.334**		
9. SE	0.047	0.001	0.067	0.051	−0.010	0.051	0.285**	0.209**	
10. LE	0.031	0.048	0.029	0.038	0.098*	0.060	0.264**	0.488**	0.591**

**p < 0.01, *p < 0.05.

### Hypotheses testing

Research hypotheses were tested using hierarchical regression analysis. The results in Table 3 showed that (1) compared with M5, M6 showed that emotion management ability had a positive impact on college students’ learning engagement (β = 0.243, *p* < 0.001) after the influence of fixed control variables and can additionally explain the college students’ learning engagement variation of up to 9.2% (Δ*R*^2^ = 0.092). The significant term of emotion management ability offered full support for Hypothesis 1; (2) Compared with M1, M2 showed that the regression coefficient of emotion management ability was significantly positive (β = 0.239, *p* < 0.001), and an additional 10.5% (ΔR^2^ = 0.105) of psychological safety variation was explained. The results offered full support for Hypothesis 2; (3) Compared with M6, after the influence of fixed control variables and emotion management ability, psychological safety was significantly positive (β = 0.417, *p* < 0.001) and can extra explain 12.8% (ΔR^2^ = 0.128) of college students’ learning engagement, and regression coefficient between emotion management ability and college students’ learning engagement was still significant (β = 0.143, *p* < 0.01), indicating that psychological safety played a partial mediating role between emotion management ability and college students’ learning engagement. These results provided support for Hypothesis 3; (4) Compared with M3, M4 showed that the regression coefficient of emotion management ability was significantly positive (β = 0.158, *p* < 0.001), and an additional 6.5% (ΔR^2^ = 0.065) of self-efficacy variation was explained. The results offered full support for Hypothesis 4; (5) M4, 6, and 8 showed that after the influence of fixed control variables and emotion management ability, self-efficacy was significantly positive (β = 0.574, *p* < 0.001) and can extra explain 20% (ΔR^2^ = 0.200) of college students’ learning engagement, and regression coefficient between emotion management ability and college students’ learning engagement was still significant (β = 0.151, *p* < 0.001), indicating that self-efficacy played a partial mediating role between emotion management ability and college students’ learning engagement. These results provided support for Hypothesis 5.

**TABLE 3:** Results of hierarchical regression analysis.

Variables	Psychological safety		Self-efficacy		Learning engagement			
	M1	M2	M3	M4	M5	M6	M7	M8
Gender	−0.033	−0.095	0.137	0.097	0.347**	0.285**	0.324***	0.236**
Grades	0.023	0.038	−0.056	−0.046	−0.030	−0.014	−0.030	0.020
Hometown	0.103	0.119	0.226***	0.237***	0.135	0.152	0.102	0.035
Family background	0.146	0.153*	0.158*	0.162**	0.154	0.160*	0.097	0.056
Personality	0.099	0.094	0.082	0.079	0.106	0.101	0.093	0.088
Health condition	0.076	0.072	0.063	0.061	0.103	0.099	0.072	0.069
EMA		0.239***		0.158***		0.243***	0.143**	0.151***
PS							0.417***	
SE								0.574***
R^2^	0.032	0.137	0.066	0.131	0.057	0.149	0.276	0.349
ΔR^2^	0.032	0.105	0.066	0.065	0.057	0.092	0.128	0.200
*F*	2.596*	9.838***	5.494***	9.306***	4.658**	10.793***	19.610***	27.490***

***p < 0.001, **p < 0.01, *p < 0.05.

To further test the mediation effect of psychological safety and self-efficacy, we used the procedures proposed by Preacher and Hayes (2004) and Preacher and Hayes (2008) and applied bias-corrected bootstrapping method to further examine the mediation effect through the “Process” plugin of SPSS 25.0. This method can produce higher statistical power. The bootstrapping sample size was set to 5,000, the confidence interval was set to 95%, and the results were shown in Table 4.

**TABLE 4:** Results of bootstrapping mediation effect examination.

Paths	Effect	SE	LLCI	ULCI
Emotion management ability→psychological safety→learning engagement	0.100	0.024	0.057	0.150
Emotion management ability→self-efficacy→learning engagement	0.092	0.025	0.047	0.144

The bootstrapping mediation analysis showed that at the 95% confidence interval level, (1) the indirect effect of psychological safety between emotion management ability and college students’ learning engagement was 0.100 and the confidence interval (LLCI = 0.057, ULCI = 0.150) did not included 0, indicating that Hypothesis 3 got full supported. (2) the indirect effect of self-efficacy between emotion management ability and college students’ learning engagement was 0.092 and the confidence interval (LLCI = 0.047, ULCI = 0.144) did not include 0, indicating that Hypothesis 5 got full supported.

## Discussion

The current study explores the relationship between emotion management ability and learning engagement. The findings show that emotion management ability can affect college students’ learning engagement through two indirect paths: emotion management ability affects college students’ learning engagement by improving their psychological safety; emotion management ability enhances college students’ self-efficacy, which in turn improving their learning engagement. Thus, improving college students’ emotion management ability is helpful to enhance their learning engagement, and improving college students’ sense of psychological safety and self-efficacy is also helpful to enhance their learning engagement.

### Suggestions

Based on the findings of the study, the current study argues that the overall level of college students’ learning engagement can be improved by regulating their emotion management ability, enhancing their psychological safety, and promoting their self-efficacy.

First, regulate college students’ emotion management ability to improve their learning engagement. College students with higher emotion management ability will actively manage their own emotion and thus proactively and positively adjust their behaviors. This study also found a positive effect of emotion management ability on college students’ learning engagement. From the perspective of emotion management ability, the present study proposes suggestions from three aspects: university, family and individual. (1) University aspect. Universities can improve the following two aspects in order to enhance the level of college students’ learning engagement. ➀ The reform of China’s education system should integrate the cultivation of emotion management ability into college education and teaching to enhance the level of college students’ emotion management ability and promote the improvement of their learning engagement. On the one hand, universities can expand the scope of curriculum selection, enrich the curriculum system, enhance the flexibility of the curriculum, and set the requirements and goals for the cultivation of college students’ emotional management ability, so that education on emotion management ability gradually penetrates into daily education and teaching activities, and let college students gradually understand the importance of emotion management ability, and guide them to receive education and cultivation in this regard in the process of curriculum selection. On the other hand, universities can also establish a combination of assessment and counseling mechanisms to reduce the emotional and psychological stress of college students, so as to enhance their enthusiasm and initiative for learning. The establishment of assessment and counseling system can help universities to grasp the problems of college students in emotion management ability, so that they can summarize them in various aspects, and then propose solutions to give more help to college students, such as conducting targeted lectures, quality development training, and training activities about emotional management ability. ➁ Improve the emotion management ability of university teachers. College students have a lot of contact with lecturers and counselors during their school years, and every word and action of teachers will influence them. Especially for freshmen who have just entered university, their minds are immature and need guidance from university teachers. Therefore, university teachers need to improve their own emotion management ability and set an example for college students, so that they can learn how to manage their emotion from teachers and better participate in learning activities. In addition, counselors also need to take the initiative to pay attention to college students’ study condition and mental health, strengthen communication, and provide timely guidance and education to students who are confused. (2) Family aspect. Parents need to maintain good communication with college students and give them a warm and comfortable environment to grow up. Parents have a significant influence on their children’s emotion management ability. Parents should always communicate with their children, pay attention to the things, confusion, and stress they encounter in university, understand children’s ideas, and give them support. At the same time, parents also should guide their children to face things positively and optimistically and deal with stress, so as to enhance their active engagement in life and learning. (3) College students aspect. College students need to learn to actively monitor and regulate their own emotion. For example, they can achieve these goals through physical and mental relaxation, strengthening physical exercise, reasonable venting of emotion, and doing things they are interested in to divert their attention and transform their negative emotion. Thus, they can keep a clear head and deal with them calmly when facing stress and difficulties. In addition, college students can also improve their own emotion management ability by building their own interpersonal network and developing the ability to communicate with others.

Second, enhance college students’ psychological safety to improve their learning engagement. (1) University aspect. Teachers play a significant role in the growth process of college students, especially in enhancing their sense of psychological safety. A supportive instructional style can effectively control the negative emotion and negative behaviors of college students, which is a basic guarantee for improving their sense of psychological safety. A supportive instructional style can give students effective feedback and resource support in a timely manner, encourage students to make their own judgments, expand their learning scope, and stimulate their interest in learning and their learning initiative. At the same time, teachers also need to pay attention to emotional care and psychological comfort for college students, enhance their sense of psychological safety, increase opportunities for communication and exchange with college students, maintain sensitivity to students’ psychological states, take the initiative to understand the problems they encounter and the psychological stressors they face, and take appropriate measures to help students relieve stress and improve their sense of psychological safety, so as to better promote their learning engagement. (2) Family aspect. Parents need to give college students enough care and not make them feel that they need to decide everything by themselves after they leave their parents and enter the college campus, and become alone. Meanwhile, parents also need to respect their children’s opinions and give them guidance when they need it, so that their minds are in a more stable state. Parents can use children’s summer and winter vacations to strengthen their emotional interaction with their children and put themselves in their shoes to make their children feel secure enough. (3) College students aspect. College students need to face their hearts and analyze the reasons for their psychological insecurity and face them squarely. They also should develop hobbies, expand social circles, and change themselves to live a more fulfilling and happy life.

Finally, promote college students’ self-efficacy to enhance their learning engagement. (1) University aspect. Universities should strengthen the professional self-efficacy of college students, pay attention to the construction of school subjects, and let universities have more academic resources and employment learning opportunities, so as to enhance students’ subject self-efficacy. Teachers need to guide students to clarify their own learning and development goals, have a good perception of themselves, improve their learning ability, develop good learning habits, and enhance their passion and love for learning. Teachers also need to encourage students and raise college students’ expectations of their own abilities; such expectations will motivate them to change their own behaviors and ways of thinking to better engage in their learning. (2) Family aspect. Parents need to help college students learn to cope with setbacks, face failures and learn from their experiences. When college students face setbacks and difficulties and cannot adjust themselves in time, their self-efficacy will become low and their self-confidence will take a serious blow. Parents need to empathize with their children and empathize with them from the heart, so that they can feel the power of comfort. Parents also need to set an example for college students, which means that when parents face failure or setbacks they need to face them with optimism, cope with them strongly, and maintain their self-confidence, thus exerting a subtle influence on their children. (3) College students aspect. College students need to realize that the improvement of self-efficacy needs to start from themselves. They should continuously improve their own quality, study hard to learn professional and cultural knowledge, cultivate hobbies, have an objective evaluation of their abilities, make reasonable plans, learn to face failures and setbacks with a reasonable mindset, learn to attribute correctly, and integrate into the collective and work together with team members to improve the sense of collective efficacy.

### Limitations and future research

Our study has several limitations. First, we finally returned 580 valid surveys. The main subjects of the study are college students in universities, while the number of Chinese universities is large and the types are different. The present study is constrained by the subjective and objective conditions, only 650 students from four universities in northern China were selected for the study, resulting in an inadequate sample size, which leads to some limitations of the research findings. Second, the surveys in our study were mainly self-assessed by new generation employees. Although the Harmon’ one-factor test was used to verify that there was no serious common method bias, it was not possible to completely exclude the possibility of its existence. Future research can used a staged data collection approach to weaken common method bias. Third, only six possible control variables were selected in conjunction with previous studies in this study, these are still incomplete. For example, we did not examine whether college students had served as student leaders. Finally, this study was not comprehensive in examining the antecedent variables of college students’ learning engagement. We only examined the effects of emotion management ability, psychological safety, and self-efficacy on learning engagement and there could be other variables between emotion management ability and learning engagement, such as social support.

## Data availability statement

The original contributions presented in the study are included in the article/supplementary material, further inquiries can be directed to the corresponding author.

## Author contributions

XL made significant contributions to the study concept and design, and was primarily responsible for designing the study, collecting and analyzing data, drafting the manuscript, making several revisions and refinements to the content of the manuscript.
